# Effect of Variable Priority Cognitive-Motor Dual-Task Training on Cognitive and Physical Function in Older Adults: A Systematic Review

**DOI:** 10.3390/brainsci16030308

**Published:** 2026-03-13

**Authors:** Xiao Yu, Roxana Dev Omar Dev, Maizatul Mardiana Harun

**Affiliations:** 1 Department of Sports Studies, Universiti Putra Malaysia UPM, Serdang 43400, Malaysia; yuxiao133968@gmail.com; 2 Department of Counsellor Education and Counselling, Universiti Putra Malaysia UPM, Serdang 43400, Malaysia; maizatul.harun@upm.edu.my

**Keywords:** variable-priority, dual-task training, balance, mobility, gait, older adults

## Abstract

**Highlights:**

**What are the main findings?**

- Variable-priority cognitive-motor dual-task training is generally associated with improvements in functional balance and mobility in older adults, but superiority over fixed-priority dual-task training is inconsistent across trials.
- Cognitive and psychosocial outcomes are rarely assessed; limited evidence suggests potential benefits for processing speed and fall-related confidence in longer programs.

**What are the implications of the main findings?**

- Future variable-priority cognitive-motor dual-task training should standardize prioritization instructions and dual-task outcome reporting (including dual-task costs) to enable synthesis and translation.
- Clinicians may consider variable-priority cognitive-motor dual-task training to train real-world mobility under attentional competition, but should not assume it is universally superior to fixed-priority dual-task training.

**Abstract:**

**Background:** With advancing age, cognitive control and postural-gait regulation decline, while dual-task interference intensifies, leading to restricted mobility and increased fall risk. Variable-priority cognitive-motor dual-task training (VPDT) enhances attentional flexibility and task integration by systematically shifting attentional allocation during training. However, its effects on cognitive and physical function remain unclear. **Objective:** To review the effects of VPDT on cognitive and physical function in older adults. **Method:** A comprehensive database search was conducted in the PubMed, Embase, Cochrane, Web of Science, PsycInfo, and CINAHL databases from inception to April 2025, relevant articles were selected, data were extracted using a PICO framework and synthesized narratively. **Result:** Eight controlled trials (*n* = 284) were included. Across studies, VPDT was generally associated with improvements in functional balance and mobility outcomes, while between-group differences versus fixed-priority dual-task training (FPDT) were inconsistent. Cognitive outcomes were sparsely reported (only one trial), and psychosocial outcomes were assessed in only a small subset of studies, precluding firm inferences regarding cognitive or psychosocial benefits. Overall risk of bias was predominantly “some concerns,” with two studies rated “high risk,” and overall certainty of evidence ranged from low to moderate due to risk of bias, small samples, and heterogeneity in protocols and outcomes. **Conclusions:** VPDT may improve physical function in older adults, particularly balance and mobility, but current evidence does not demonstrate a consistent incremental advantage over FPDT. Confidence in comparative effects remains limited due to small sample sizes, risk-of-bias concerns, and heterogeneity in intervention design and outcome measurement.

## 1. Introduction

Falls and reduced mobility represent one of the most prominent public health issues among the elderly population, frequently leading to fractures, functional decline, disability, and diminished quality of life, while imposing a significant burden on healthcare and caregiving resources [1]. In community settings, the annual prevalence of falls in adults aged ≥65 years is commonly reported to be around one-quarter to one-third, increasing further with advanced age [2]. The World Health Organization notes that falls pose a substantial risk of injury and death globally, occurring particularly frequently during the later stages of life [3].

To slow the decline in physical function among older adults, exercise interventions have long been regarded as a cornerstone strategy [4]. However, research indicates that exercise interventions alone can only reduce fall risk by approximately 16% [5]. In real-life settings, older adults’ mobility is often accompanied by concurrent cognitive activities. For younger individuals, walking is typically an automated process requiring minimal attentional resources. However, due to degenerative changes in the nervous system, walking in older adults becomes increasingly dependent on conscious cognitive control [6]. When cognitive resources (attentional resources) are overtaxed by concurrent tasks, gait stability significantly declines, progressively increasing fall risk [7]. When individuals must perform two tasks simultaneously, attentional resources are divided, potentially compromising gait stability and increasing fall risk. This phenomenon, often referred to as dual-task interference, is particularly pronounced in older adults because age-related declines in executive control and attentional regulation reduce the ability to flexibly allocate cognitive resources during locomotion [8]. Given the complex interactions between motor and cognitive systems in maintaining balance and gait control, developing effective interventions to enhance dual-task processing in older adults is urgently needed.

Numerous reviews have demonstrated the efficacy of cognitive-motor dual-task training in enhancing cognitive and physical function in older adults [9]. This intervention approach, closely aligned with daily functional contexts, focuses not solely on improving muscle strength or balance. Instead, it strengthens coordination and resource allocation capabilities under dual-task conditions by simultaneously challenging motor control and cognitive control during training [10]. Dual-task training is typically categorized into two types: fixed-priority cognitive-motor dual-task training (FPDT) and variable-priority cognitive-motor dual-task training (VPDT). FPDT requires participants to maintain a constant allocation of attention between cognitive and motor tasks throughout the intervention; whereas VPDT requires participants to consciously shift their attentional focus from cognitive to motor tasks and vice versa at different stages according to instructions [11]. Older adults increasingly rely on executive attention to maintain gait and balance, especially under dual-task conditions, and neuroimaging evidence consistently shows elevated prefrontal cortex (PFC) activation during dual-task mobility, reflecting higher attentional resource demands [12,13]. Building on this, VPDT is designed to train flexible attentional allocation by systematically shifting task emphasis between the motor and cognitive components, thereby targeting task-set switching and reducing cognitive–motor interference. Consistent with this rationale, variable-priority strategy training in older adults has demonstrated transfer effects to untrained dual-task contexts, suggesting that how attention is instructed and re-weighted can be an active ingredient of adaptation [14]. Moreover, dual-task training has been linked to concurrent changes in task-related brain activation alongside improvements in dual-task walking, supporting an experience-dependent neuroplasticity account [15].

Despite widespread recognition of cognitive-motor dual-task training, debate persists regarding which priority strategy yields superior outcomes. Some scholars contend that VPDT is highly effective in enhancing “attentional flexibility.” By dynamically shifting focus between tasks, VPDT may help older adults develop more efficient resource allocation patterns, thereby demonstrating better transfer effects in dynamic living environments [14]. However, existing randomized controlled trial results are not entirely consistent. Some studies indicate VPDT significantly improves gait speed but shows little difference from FPDT in long-term maintenance of cognitive function [16,17]. While the current literature reviews examine dual-task training’s overall efficacy, systematic evaluations specifically targeting the “variable priority” training paradigm remain relatively scarce. Most existing reviews lump different priority strategies into a single category, hindering precise clinical guidance. Moreover, direct empirical support for this mechanism in older adults remains limited because most trials emphasize physical endpoints and only a small subset includes standardized cognitive measures. Accordingly, the present review evaluates VPDT effects on physical and cognitive outcomes while explicitly mapping the evidence gaps relevant to proposed cognitive–motor mechanisms.

Therefore, this systematic review aims to comprehensively analyze the effects of VPDT on cognitive and physical function in older adults. It further evaluates the efficacy of VPDT in enhancing gait and cognitive function while exploring optimal intervention combinations, thereby providing evidence for fall prevention interventions and cognitive maintenance in this population.

## 2. Methodology

### 2.1. Protocol and Registration

This article was registered on PROSPERO (CRD42024503555) and conducted following the Preferred Reporting Items for Systematic Reviews and Meta-Analyses (PRISMA 2020) guidelines. The completed PRISMA 2020 checklist is provided in the Supplementary Materials.

### 2.2. Eligibility Criteria

Studies were selected based on the following pre-defined PICOS criteria (see Table 1). We included only peer-reviewed, full-text articles published in English, and retrievable in full text through institutional subscriptions, open-access sources, or author contact. Conference abstracts, posters, editorials, commentaries, protocols without results, and the non-peer-reviewed gray literature were excluded.

### 2.3. Search Strategy

A comprehensive search was conducted from inception to April 2025 in the following sources: PubMed, Embase, Cochrane, Web of Science, PsycInfo, CINAHL, and Google Scholar. Search strategies combined MeSH and entry terms including (“dual task*” OR “dual-task*” OR “motor-cognitive” OR “cognitive-motor”) AND (“variable priorit*” OR “variable-priority” OR “priority instruction*” OR “attention allocation” OR “attentional shift*” OR “task switching”) AND (training OR intervention OR exercise OR program*) AND (“older adult*” OR elderly OR ageing OR aging). In addition, we manually screened the reference lists of all included studies and relevant systematic reviews. Google Scholar was also used as an additional search source to ensure that no potential eligible studies were missed. Results were sorted by keywords relevance articles, and the first 200 records were screened. Reference lists of relevant studies identified through Google Scholar were also examined to identify additional eligible articles.

### 2.4. Study Selection

The literature was initially searched by two independent researchers using the same retrieval strategy. Databases were searched, and the retrieved literature was imported into Zotero (7.0.15) reference management software, and duplicates were removed. Titles and abstracts were screened independently by two reviewers; any record deemed potentially eligible by either reviewer was carried forward to full-text retrieval and eligibility assessment. Full-text eligibility was then assessed independently by both reviewers for the same set of reports. Disagreements were resolved by consensus discussion, with adjudication by a third reviewer (RO) when necessary. Inter-rater agreement prior to consensus was quantified using Cohen’s kappa (κ) for both the title/abstract screening stage and the full-text eligibility stage.

### 2.5. Data Extraction

Data extraction included the following: (1) author names and publication year; (2) number of participants, gender, age, and health status; (3) intervention method, duration in weeks, frequency, and duration; (4) outcome measures. Two researchers independently extracted data, which were then pooled. In cases of disagreement between the two researchers, a third researcher (RO) resolved the discrepancy to reach a consensus.

### 2.6. Quality Assessment

The methodological quality of each RCT was assessed using the Cochrane Risk of Bias Tool (RoB 2.0) [18]. We evaluated five specific domains: bias arising from the randomization process; bias due to deviations from intended interventions; bias due to missing outcome data; bias in measurement of the outcome; bias in selection of the reported result [19]. Each domain was categorized as “low risk,” “some concerns,” or “high risk.”

### 2.7. Data Synthesis and Analysis

All outcomes were summarized in evidence tables. For each study, we reported the intervention/comparator, assessment timepoints, and results as presented by the original authors. Meta-analysis was not performed because substantial clinical and methodological heterogeneity was observed across the included trials, including differences in participant characteristics, intervention dose and content, comparator conditions, and outcome definitions/reporting. In addition, several outcomes were reported using non-comparable metrics or insufficient summary statistics, which further limited effect-size pooling. We synthesized findings narratively using a structured approach consistent with the SWiM reporting guideline, focusing on consistency in direction of effects and clinical relevance across studies [20]. Risk of bias for randomized trials was assessed using RoB 2 and incorporated into interpretation [19]. The certainty of evidence for key outcome domains was rated using the GRADE approach [21] and summarized in a Supplementary Summary of Findings table (Supplementary Table S1).

## 3. Results

### 3.1. Risk of Bias and Certainty of Evidence Assessment

**Figure 1:**
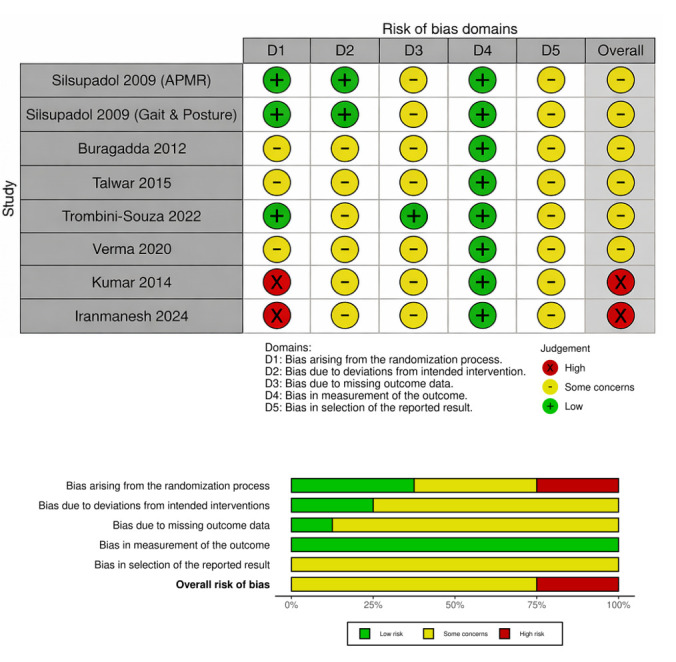
Risk of bias graph and summary [16,17,22,23,24,25,26,27].

Across the eight included trials, overall risk of bias was mainly “some concerns,” with two studies rated as high risk (Figure 1). The domain measurement of outcomes (D4) was low risk in all studies, reflecting the use of standardized balance, mobility, and cognitive tests. In contrast, selection of the reported result (D5) raised some concerns in all studies, largely due to limited availability of trial registration/protocols. Randomization (D1) was the main source of high risk in two studies, while deviations from intended interventions (D2) and missing outcome data (D3) were commonly judged as some concerns because reporting of adherence, blinding, and attrition handling was often incomplete. According to the GRADE assessment, the certainty of evidence ranged from moderate for functional balance outcomes to low for functional mobility, cognitive, and psychosocial outcomes (Supplementary Table S1). Certainty was downgraded primarily for risk of bias and imprecision (small samples and heterogeneous protocols/outcomes), with additional concerns about inconsistency across comparators and populations in some outcomes.

### 3.2. Study Selection and Characteristics

**Figure 2:**
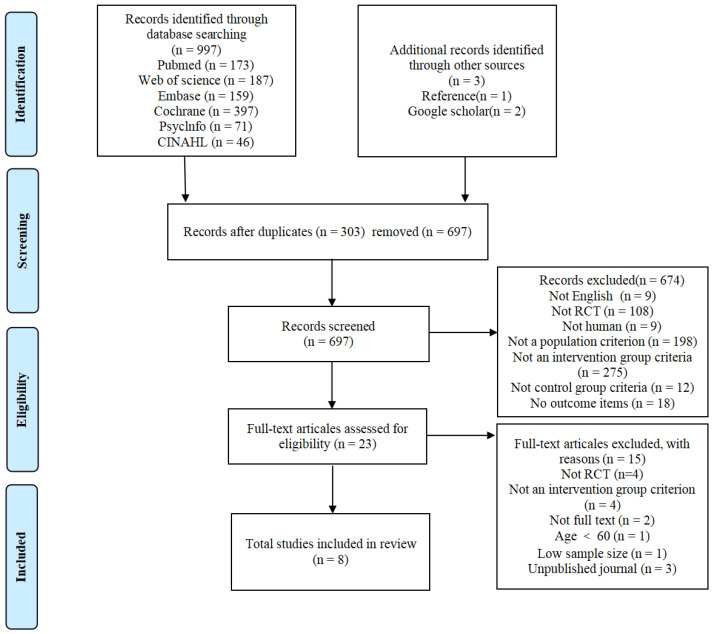
PRISMA flow chart for systematic review.

As shown in Figure 2, the study selection process followed PRISMA 2020. At the title/abstract screening stage (*n* = 697), Reviewer 1 and Reviewer 2 initially identified 23 and 17 records, respectively, as potentially eligible. There were six discrepant decisions at this stage, which were resolved by consensus. Cohen’s kappa indicated almost perfect agreement (κ = 0.846; percentage agreement = 99.1%) prior to reconciliation. At the full-text eligibility stage (*n* = 23), both reviewers independently assessed the same set of full-text reports and reached perfect agreement on inclusion decisions (κ = 1.00; percentage agreement = 100%), resulting in eight studies included in the final synthesis.

Following reconciliation, a total of eight studies met all inclusion criteria and were included in this review (Table 2). Among these, Silsupadol et al. [17] and Silsupadol et al. [16] reported different outcomes (balance ability and gait parameters) from the same randomized controlled trial; therefore, they were considered two separate pieces of evidence but counted as a single study in the total sample size. The pooled sample comprised 284 older adults (mean age ≥ 60 years). Study populations included community-dwelling healthy older adults, individuals with balance disorders, and institutionalized older adults. The intervention group received VPDT, while control groups received FPDT, ST, or usual care. Intervention durations varied considerably (2–24 weeks; typically 45–60 min/session, 2–5 sessions/week). Outcomes primarily focused on balance and mobility, including dual-task gait performance and balance confidence. A minority of studies assessed cognitive processing and psychosocial outcomes.

### 3.3. Effects on Physical Function

All included studies assessed physical outcomes, most commonly BBS and/or TUG. Because comparator conditions varied across trials, we summarize findings using VPDT versus FPDT (head-to-head comparisons), and dual-task training (VPDT/FPDT) versus single-task training/usual care (when a non-dual-task comparator was included). Overall, dual-task training tended to outperform single-task/usual care on functional balance and mobility outcomes. However, head-to-head comparisons between VPDT and FPDT were mixed, and the current evidence does not show a consistent incremental advantage of VPDT over FPDT across outcomes.

Regarding balance ability, six studies employed BBS. Silsupadol et al. demonstrated that dual-task training (FPDT/VPDT) yielded greater overall balance-related improvements compared to single-task training, suggesting VPDT may hold an advantage in enhancing “dual-task-related performance” [16,17]. In direct comparisons between VPDT and FPDT, Buragadda et al. reported improvements in both groups, but between-group comparisons showed VPDT superior to FPDT on the BBS and ABC (balance confidence) (*p* < 0.05) [22]. Similarly, Kumar et al. demonstrated improvements in both groups, but VPDT showed “more pronounced” gains (*p* < 0.001) [24]. In contrast, Talwar et al. showed significant within-group improvements in BBS and DGI across all three groups, but no significant between-group differences in these measures post-intervention (*p* > 0.05) [25]. Iranmanesh et al. also reported BBS improvements in all groups, yet balance outcomes showed no significant between-group differences (*p* > 0.05) [23].

Regarding mobility and dual-task mobility, three studies examined the TUG, and one studied the DT-TUG. In Talwar et al. all groups improved on the TUG (*p* = 0.001); intergroup differences existed on the DT-TUG, with further pairwise comparisons showing both dual-task training groups outperformed the single-task group (*p* = 0.001), though no significant difference existed between VPDT and FPDT (*p* > 0.05) [25]. Verma et al. also reported that dual-task training groups generally showed greater improvements in BBS and TUG than single-task groups (*p* < 0.05) [27].

Overall, compared to single-task training, dual-task training (FPDT/VPDT) is more likely to improve balance-related function and mobility. However, the advantage of VPDT over FPDT is inconsistent: some studies reported statistically significant between-group differences favoring VPDT on selected balance-related outcomes, whereas other studies reported no significant between-group differences between VPDT and FPDT. The VPDT–FPDT comparisons did not point in a single direction across outcomes, and this inconsistency likely reflects, at least in part, differences in study quality and in how comparable the trials were. As summarized in the risk-of-bias assessment, several trials had “some concerns” or “high risk” judgments, reflecting issues such as incomplete reporting of randomization procedures, potential deviations from intended interventions, and selective outcome reporting. In addition, trials differed substantially in intervention dose and content (e.g., duration, weekly frequency, and the specific cognitive–motor task combinations trained) as well as in outcome definitions and reporting formats. These limitations likely increased imprecision and reduced the ability to detect consistent between-group differences, thereby contributing to the observed variability in VPDT-versus-FPDT effects.

### 3.4. Effects on Cognitive Outcomes

Only one study reported cognitive outcomes, and thus the evidence for cognitive benefits of VPDT remains preliminary and should be interpreted cautiously. Iranmanesh et al. employed the serial reaction time task as a cognitive metric [23]. Results indicated that training modality significantly influenced reaction times, with both dual-task training groups outperforming the single-task group (*p* < 0.05). In addition, VPDT reaction times were significantly lower than FPDT (*p* < 0.05), suggesting variable priority directives may exert an additional facilitative effect on information processing speed. Given the extremely limited reporting of cognitive outcomes, robust conclusions regarding the cognitive benefits of VPDT remain challenging at present.

### 3.5. Effects on Psychosocial Outcomes and Quality of Life

Psychosocial outcomes were evaluated in only one or two trials, which limits the strength of inference and precludes firm conclusions in this domain. Trombini-Souza et al. found no between-group differences (progression from VP-to-FP vs. VP-only) [26]. However, significant time effects indicated improvements across several patient-reported endpoints, including reduced concern about falling (MD = −2.91) and depression symptoms (MD = −1.66), alongside increases in health-related quality of life domains (physical function MD = 7.86; overall mental health MD = 5.82; vitality MD = 10.45; general health MD = 6.81; perceived health compared to last year MD = 11.89). Overall, evidence linking psychological outcomes to quality of life remains insufficient and limited, and should be interpreted with caution.

## 4. Discussion

### 4.1. Principal Findings

This systematic review included eight controlled trials involving participants aged ≥60 years (including those with mild cognitive impairment and cognitively intact individuals) to evaluate the effects of VPDT on cognitive and physical function. The included studies suggest that VPDT is associated with improvements in physical function (particularly balance and mobility). However, whether VPDT provides incremental benefit over FPDT remains unclear, as direct comparisons were inconsistent across studies and confidence is limited by risk of bias, small sample sizes, and protocol heterogeneity. In the few trials assessing psychosocial outcomes, dual-task training may also improve fall-related psychological indicators and certain health outcomes. Methodologically, most studies exhibited a risk of bias categorized as “some concerns,” and the certainty of evidence was predominantly low to moderate.

### 4.2. Physical Function Outcomes

Our findings align with the “task integration hypothesis.” This hypothesis posits that practicing coordination between two tasks is more effective than practicing each task separately or maintaining fixed attention [16,17]. Regarding physical function, VPDT improvements were most consistently observed in measures related to functional balance and mobility, consistent with other reviews [28,29]. The mechanism lies in VPDT compelling the central nervous system (CNS) to develop more flexible postural control strategies. By explicitly directing participants to shift attention between stability and cognitive tasks, VPDT may circumvent the “postural primacy strategy” typically defaulted to by older adults, thereby training them to maintain balance even when cognitive resources are diverted [6,8].

Furthermore, Silsupadol et al. observed that VPDT also improved performance on untrained dual-tasks compared to ST and FPDT, a phenomenon termed the transfer effect [16,17]. This arises from enhanced adaptability in brain attention when shifting between tasks, enabling these skills to transfer to other, untrained yet similar dual-task environments. Consequently, older adults may learn more rapidly in the VPDT group [30].

However, findings on VPDT versus FPDT are inconsistent. Some studies indicate more pronounced improvements with variable priority, while others show comparable gains with both protocols. This variability may relate to task type, intervention design, training dose, baseline levels, and outcome measures. Therefore, this study concludes that priority strategies possess theoretical validity and practical applicability. However, the current number of trials remains insufficient and their quality limited, making it difficult to definitively conclude that “VPDT is clearly superior.”

### 4.3. Cognitive Outcomes

Reports on cognitive outcomes were even less consistent, with considerable variation in measurement tools and sensitivity. Only one study reported improvements in cognitive function, suggesting that practicing “allocating/switching attentional resources between motor and cognitive tasks” may exert some promotional effect on cognitive control processes [23]. However, it is crucial to emphasize that the cognitive evidence suffers from small sample sizes and inadequate reporting. These factors reduce precision and increase the risk of bias, thereby diminishing the certainty of the evidence [19,21]. Therefore, any statements regarding cognitive benefits of VPDT should be interpreted as preliminary and primarily serve to highlight an evidence gap rather than to support firm conclusions.

### 4.4. Psychosocial Outcomes and Quality of Life

Psychosocial indicators (such as balance confidence, fall concern, depressive symptoms, and quality of life), though reported in fewer studies (only two), hold significant clinical importance as they influence activity participation and long-term adherence. Buragadda et al. reported significantly higher scores on the ABC scale for VPDT compared to FPDT, suggesting that mastering attention allocation may translate into subjective confidence during daily activities [22]. Trombini-Souza et al. also assessed the Fall Efficacy Scale-International (FES-I) and quality of life measures (SF-36) [26]. Although both groups showed significant reductions in fall fear and depressive symptoms over time, no significant interaction was found between the VPDT and FPDT groups. These findings suggest that the decrease in fall fear may stem from participants’ general exposure to dual-task challenges and increased physical activity, rather than the specific variable priority strategy.

### 4.5. Limitations

The main limitations of the evidence in this review relate to both internal validity and precision. Several trials had risk-of-bias concerns due to incomplete reporting of key methodological procedures (e.g., randomization and allocation processes) and potential issues related to intervention adherence and selective reporting, most outcomes are likely underpowered for detecting small-to-moderate between-group differences, which contributes to imprecision and uncertainty in comparative inferences. Cognitive outcomes were sparsely reported (only one trial), and the lack of consistent cognitive measures and follow-up time points further limited inference regarding cognitive effects. Furthermore, the relatively small overall sample size, together with substantial clinical and methodological heterogeneity, limited the feasibility and interpretability of a meta-analysis. Specifically, intervention dose varied markedly (2–24 weeks; 2–5 sessions/week; 45–60 min/session), comparator conditions differed (FPDT vs. single-task training/usual care), and outcome measures and reporting formats were not sufficiently consistent to support robust pooling; in addition, several trials did not provide compatible summary statistics (e.g., SD/SE for change scores or comparable post-intervention time points) required for effect-size calculation.

## 5. Conclusions

This systematic review included eight controlled trials to comprehensively evaluate the effects of VPDT in older adults aged 60 and over. Overall, dual-task training with clearly defined task prioritization instructions was associated with improvements in functional balance and mobility, particularly in outcome measures reflecting attentional load. However, the advantage of VPDT over FPDT is not consistent; existing evidence is limited by heterogeneity in training protocols and outcome measures, small sample sizes, and risk of bias primarily attributed to “some concerns.” Cognitive and psychosocial outcome reporting is inadequate and inconsistent, because these domains were assessed in only a small number of trials; while longer-term interventions showed potential benefits in balance confidence, fall anxiety, and quality of life, the certainty of the evidence remains low to moderate.

The current evidence base points to specific, correctable design and reporting gaps that should be addressed to make future VPDT trials interpretable and clinically actionable. VPDT must be treated as a parameterized intervention, studies should pre-specify and report the priority-allocation schedule as the proportion of session time spent under motor-priority versus cognitive-priority instructions (a priority “ratio”), the switching structure, and explicit progression rules (how and when task difficulty and/or switching frequency is increased). Because existing trials do not provide convergent evidence to recommend a single optimal ratio, future studies should prospectively compare candidate schedules and examine whether baseline balance/cognitive status moderates response. Moreover, to directly test the hypothesized cognitive–motor mechanism and to reduce heterogeneity, adequately powered, preregistered head-to-head VPDT versus FPDT trials with blinded outcome assessment should adopt a core outcome set and harmonized time points (baseline, post-intervention, and follow-up). Fidelity indicators (attendance, adherence to priority instructions, and cognitive-task accuracy during training) should be routinely documented to ensure the intended priority manipulation is actually delivered. These steps would allow future trials to determine whether VPDT provides a reproducible incremental benefit over FPDT and to identify priority-setting strategies that can be recommended with greater certainty.

## Figures and Tables

**Table 1:** Eligibility criteria for research articles.

Criteria	Description
Population (P)	Older adults aged ≥60 years, including cognitively impaired or unimpaired populations; both clinical and non-clinical samples were eligible.
Intervention (I)	Variable-priority cognitive–motor dual-task training: simultaneous motor + cognitive task practice with systematic switching of attentional priority between tasks across trials/blocks/sessions.
Comparison (C)	Any comparator/control group.
Outcome (O)	At least one cognitive or one physical/functional outcome (e.g., executive function/attention/working memory; gait, balance, mobility such as TUG; falls-related outcomes).
Study Design (S)	Controlled trials with a comparator group, including randomized controlled trials (parallel, cluster, crossover, pilot/feasibility randomized trials).
Inclusion Criteria	Studies meeting all PICOS elements above and reporting baseline and post-intervention data (or change scores) for ≥1 eligible outcome.
Exclusion Criteria	Not older adults or no extractable older-adult data; not VPDT group; no comparator group; no eligible outcomes; insufficient data for extraction.

**Table 2:** Table of data extraction (N = 8).

Study	Design	Participants	Groups	Intervention	Dose	Outcomes	Main Finding
Silsupadol et al. [17]	Double-blind randomized controlled trial	Balance impairment; *n* = 23; age ≥ 65; (ST = 7, FPDT = 8, VPDT = 6)	ST vs. FPDT vs. VPDT	Task-specific balance training; FPDT = equal attention; VPDT = alternate attention between postural vs. secondary blocks; participants and assessors blinded to group identity	45 min, 3/week, 4 weeks	BBS, ABC, chair-stand/turning task, TUG	DT groups improved more than ST; VPDT tended to yield better DT-related
Silsupadol et al. [16]	Double-blind randomized controlled trial	Balance impairment; *n* = 23; age ≥ 65; (ST = 7, FPDT = 8, VPDT = 6)	ST vs. FPDT vs. VPDT	FPDT = equal emphasis; VPDT = alternate emphasis between tasks in blocks	45 min, 3/week, 4 weeks	Gait under DT (speed/accuracy)	DT training improved DT-walking vs. ST; VPDT often shows better transfer to DT contexts
Buragadda et al. [22]	Controlled trial	Balance impairment; *n* = 30; age ≥ 65; (FPDT = 15, VPDT = 15)	FPDT vs. VPDT	Dual-task balance training; FPDT vs. VPDT instructional sets	45 min, 3/week, 4 weeks	BBS, ABC (pre/post)	Both improved; VPDT > FPDT on BBS and ABC between groups
Talwar et al. [25]	Randomized controlled trial	Balance impairment; *n* = 60; age ≥ 65; (ST = 20, FPDT = 20, VPDT = 20)	ST vs. FPDT vs. VPDT	Agility/square-stepping exercise; DT with FPDT vs. VPDT instructions	1 h, 3/week, 4 weeks	BBS, DGI, TUG, DT-TUG baseline/post	Dual-task >single-task; VPDT vs. FPDT: no significant difference
Verma et al. [27]	Randomized pre–post experimental study	Balance impairment; *n* = 45; age ≥ 65; (ST = 15, FPDT = 15, VPDT = 15)	ST vs. FPDT vs. VPDT	Same balance tasks + secondary tasks; FPDT = maintain attention on both; VPDT = half focus postural, half focus secondary	1 h, 5/week, 2 weeks	BBS, TUG (pre/post)	DT groups improved more than ST
Kumar et al. [24]	Controlled pre–post test study	Balance impairment; *n* = 30; age ≥ 65; (FPDT = 15, VPDT = 15)	FPDT vs. VPDT	Dual-task balance training with FPDT vs. VPDT instruction sets	45 min, 3/week, 4 weeks	Balance	Both improved; VPDT “more marked” improvement than FPDT
Iranmanesh et al. [23]	Controlled trial	Older adults; *n* = 36; age 65–80; (control = 12, FPDT = 12, VPDT = 12)	control vs. FPDT vs. VPDT	Dual-task balance training with FPDT vs. VPDT priority	45 min, 3/week, 4 weeks	BBS, serial reaction time task	Cognition improved; VPDT showed faster information processing; balance difference not significant vs. control
Trombini-Souza et al. [26]	Randomized controlled trial	Balance impairment; *n* = 60; age 60–80; (FPDT = 30, VPDT = 30)	VPDT vs. VPDT-to-FPDT	Dual-task gait and balance training emphasizing variable attentional focus; one group progressed from VPDT to FPDT	1 h, 2/week, 24 weeks	Concern about falls, balance confidence, quality of life, depression symptoms	Both interventions improved outcomes; VPDT yielded meaningful psychosocial and balance-related benefit

Abbreviations and symbols: *n* = number; ST, single training; VPDT, variable priority dual-task training; FPDT, fixed priority dual-task training; DT, dual-task training; BBS, berg balance scale; DGI, dynamic gait index; TUG, timed up and go; DT-TUG, dual-task timed up and go; ABC, activities-specific balance confidence scale; +, exercises combinations.

## Data Availability

No new data were created or analyzed in this study. Data sharing is not applicable to this article.

## Supplementary Materials

The following supporting information can be downloaded at: https://www.mdpi.com/article/10.3390/brainsci16030308/s1, Supplementary File S1: PRISMA 2020 checklist (Reference [31] is cited in the supplementary materials). Table S1. GRADE Summary of Findings for VPDT versus FPDT in older adults.

## References

[1] Park J.H. (2021). Effects of cognitive-physical dual-task training on executive function and activity in the prefrontal cortex of older adults with mild cognitive impairment. Brain Neurorehabil..

[2] Kakara R. (2023). Nonfatal and fatal falls among adults aged ≥65 years—United States, 2020–2021. MMWR Morb. Mortal. Wkly. Rep..

[3] World Health Organization (2021). Step Safely: Strategies for Preventing and Managing Falls Across the Life-Course.

[4] Merchant R.A., Morley J.E., Izquierdo M. (2021). Exercise, aging and frailty: Guidelines for increasing function. J. Nutr. Health Aging.

[5] Choi M., Hector M. (2012). Effectiveness of intervention programs in preventing falls: A systematic review of recent 10 years and meta-analysis. J. Am. Med. Dir. Assoc..

[6] Woollacott M., Shumway-Cook A. (2002). Attention and the control of posture and gait: A review of an emerging area of research. Gait Posture.

[7] Yu X., Kamalden T.F.T., Dev R.D.O., Gasibat Q., Rani B., Dai Y., Bao L., Li J. (2023). Effects of combining physical and cognitive training on older adults’ physical performance and functional abilities: A systematic review. Int. J. Kinesiol. Sports Sci..

[8] Yogev-Seligmann G., Hausdorff J.M., Giladi N. (2008). The role of executive function and attention in gait. Mov. Disord. Off. J. Mov. Disord. Soc..

[9] Ali N., Tian H., Thabane L., Ma J., Wu H., Zhong Q., Gao Y., Sun C., Zhu Y., Wang T. (2022). The effects of dual-task training on cognitive and physical functions in older adults with cognitive impairment; a systematic review and meta-analysis. J. Prev. Alzheimer’s Dis..

[10] Varela-Vásquez L.A., Minobes-Molina E., Jerez-Roig J. (2020). Dual-task exercises in older adults: A structured review of current literature. J. Frailty Sarcopenia Falls.

[11] Lauenroth A., Ioannidis A.E., Teichmann B. (2016). Influence of combined physical and cognitive training on cognition: A systematic review. BMC Geriatr..

[12] Lapanan K., Kantha P., Nantachai G., Hemrungrojn S., Maes M. (2023). The prefrontal cortex hemodynamic responses to dual-task paradigms in older adults: A systematic review and meta-analysis. Heliyon.

[13] Li K.Z., Bherer L., Mirelman A., Maidan I., Hausdorff J.M. (2018). Cognitive involvement in balance, gait and dual-tasking in aging: A focused review from a neuroscience of aging perspective. Front. Neurol..

[14] Lussier M., Bugaiska A., Bherer L. (2017). Specific transfer effects following variable priority dual-task training in older adults. Restor. Neurol. Neurosci..

[15] Kuo H.T., Yeh N.C., Yang Y.R., Hsu W.C., Liao Y.Y., Wang R.Y. (2022). Effects of different dual task training on dual task walking and responding brain activation in older adults with mild cognitive impairment. Sci. Rep..

[16] Silsupadol P., Lugade V., Shumway-Cook A., van Donkelaar P., Chou L.S., Mayr U., Woollacott M.H. (2009). Training-related changes in dual-task walking performance of elderly persons with balance impairment: A double-blind, randomized controlled trial. Gait Posture.

[17] Silsupadol P., Shumway-Cook A., Lugade V., van Donkelaar P., Chou L.S., Mayr U., Woollacott M.H. (2009). Effects of single-task versus dual-task training on balance performance in older adults: A double-blind, randomized controlled trial. Arch. Phys. Med. Rehabil..

[18] Higgins J.P., Altman D.G. (2008). Assessing risk of bias in included studies. Cochrane Handbook for Systematic Reviews of Interventions: Cochrane Book Series.

[19] Sterne J.A.C., Savović J., Page M.J., Elbers R.G., Blencowe N.S., Boutron I., Cates C.J., Cheng H.Y., Corbett M.S., Eldridge S.M. (2019). RoB 2: A revised tool for assessing risk of bias in randomised trials. BMJ.

[20] Campbell M., McKenzie J.E., Sowden A., Katikireddi S.V., Brennan S.E., Ellis S., Hartmann-Boyce J., Ryan R., Shepperd S., Thomas J. (2020). Synthesis without meta-analysis (SWiM) in systematic reviews: Reporting guideline. BMJ.

[21] Guyatt G.H., Oxman A.D., Vist G.E., Kunz R., Falck-Ytter Y., Alonso-Coello P., Schünemann H.J. (2008). GRADE: An emerging consensus on rating quality of evidence and strength of recommendations. BMJ.

[22] Buragadda S., Alyaemni A., Melam G.R., Alghamdi M.A. (2012). Effect of dualtask training (fixed priority-versus-variable priority) for improving balance in older adults. World Appl. Sci. J..

[23] Iranmanesh H., Shirvani H., Amini A., Shamsaddini A., Sobhani V. (2024). The Effect of Different Dual-Task Balance Training Methods on Balance and Cognitive Function of Older Adults. J. Kerman Univ. Med. Sci..

[24] Kumar C. (2014). Effect of training balance under dual task with fixed and variable priority instructions with balance impairment in institutionalized elderly population. Indian J. Physiother. Occup. Ther..

[25] Talwar J., Zia N.U., Maurya M., Singh H. (2015). Effect of Agility Training Under Single-Task Condition Versus Training Under Dual-Task Condition with Different Task Priorities to Improve Balance in the Elderly. Top. Geriatr. Rehabil..

[26] Trombini-Souza F., Nogueira R.T.D.S.A., Serafim A.C.B., Lima T.M.M.D., Xavier M.K.A., Perracini M.R., de Araújo R.C., Sacco I.C., Nascimento M.D.M. (2022). Concern about falling, confidence in balance, quality of life, and depression symptoms in community-dwelling older adults after a 24-week dual-task training with variable and fixed priority: A randomized controlled trial. Res. Aging.

[27] Verma M., Awasthi S., Sharma B. (2020). To compare the effect between two different priorities dual task balance training in older adults with balance impairment. Int. J. Sci. Res. Sci. Technol..

[28] Khan M.J., Fong K.N., Wong T.W.L., Tsang W.W.N., Chen C., Chan W.C., Winser S.J. (2025). Effectiveness of dual-task exercise in improving balance and preventing falls among older adults: Systematic review with meta-analysis and meta-regression. Eur. Geriatr. Med..

[29] Yildiz S.E., Fidan O., Gulsen C., Colak E., Genc G.A. (2024). Effect of dual-task training on balance in older adults: A systematic review and meta-analysis. Arch. Gerontol. Geriatr..

[30] Kramer A.F., Larish J.F., Strayer D.L. (1995). Training for attentional control in dual task settings: A comparison of young and old adults. J. Exp. Psychol. Appl..

[31] Page M.J., McKenzie J.E., Bossuyt P.M., Boutron I., Hoffmann T.C., Mulrow C.D., Shamseer L., Tetzlaff J.M., Akl E.A., Brennan S.E. (2021). The PRISMA 2020 statement: An updated guideline for reporting systematic reviews. BMJ.

