# Supplementary material for: Effect of Variable Priority Cognitive-Motor Dual-Task Training on Cognitive and Physical Function in Older Adults: A Systematic Review
Source: Brain Sci. 2026 Mar 13;16(3):308. doi: 10.3390/brainsci16030308 (PMC13024328; doi:10.3390/brainsci16030308)
Supplement: Supplementary file 1 [file brainsci-16-00308-s001.zip › brainsci-4172967-supplementary.pdf]

Supplementary Table S1. GRADE Summary of Findings for VPDT versus FPDT in older adults

Outcome	No. of studies	Participants	Study design	Risk of bias	Inconsistency	Indirectness	Imprecision	Overall certainty (GRADE)
Functional balance (BBS)	5	194	Randomized controlled trials	Some concerns	Moderate heterogeneity	Not serious	Small sample size	Moderate
Functional mobility (TUG)	3	128	Randomized controlled trials	Some concerns	Moderate heterogeneity	Not serious	Small sample size	Low
Cognitive function	1	36	Controlled trials	Some concerns	Not applicable	Not serious	Very small sample	Low
Psychosocial outcomes (e.g., ABC scale)	2	90	Randomized controlled trials	Some concerns	Moderate heterogeneity	Not serious	Small sample	Low
